# A Growth–Survival Trade‐Off Along an Elevation Gradient Is Altered by Earthquake Disturbance in a Monodominant Southern Beech Forest

**DOI:** 10.1002/ece3.70467

**Published:** 2024-10-31

**Authors:** Robert B. Allen, Darryl I. MacKenzie, Susan K. Wiser, Peter J. Bellingham, Lawrence E. Burrows, David A. Coomes

**Affiliations:** ^1^ Independent Researcher Lincoln New Zealand; ^2^ Proteus Outram New Zealand; ^3^ Ecosystems and Conservation, Manaaki Whenua – Landcare Research Lincoln New Zealand; ^4^ Department of Plant Sciences, Conservation Research Institute University of Cambridge Cambridge UK

**Keywords:** altitude, climate change, disturbance, fast–slow continuum, *Fuscospora*, monodominant forest, New Zealand, *Nothofagus*, pace‐of‐life

## Abstract

Tree growth–survival relationships link two demographic processes that individually dictate the composition, structure and functioning of forest ecosystems. While these relationships vary intra‐specifically, it remains unclear how this reflects environmental variation and disturbance. We examined the influence of a 700‐m elevation gradient and an *Mw* = 6.7 earthquake on intra‐specific variability in growth–survival relationships. We expected that survival models that incorporated recent growth would be better supported than those only using other factors known to influence tree survival. We used a permanent plot network that representatively sampled a monodominant *Nothofagus* forest in New Zealand's Southern Alps in 1974 and that was remeasured seven times through to 2009. The relationships were assessed using pre‐earthquake growth and survival, pre‐earthquake growth and post‐earthquake survival (0–5 years post‐earthquake), and post‐earthquake growth and survival (5+ years post‐earthquake). Survival was related to growth of 4504 trees on 216 plots using Bayesian modelling. We hypothesised there would be a positive, logistic relationship between growth and survival. Pre‐earthquake, we found a positive, logarithmic growth–survival relationship at all elevations. At higher elevations, trees grew more slowly but had higher survival than trees at lower elevations, supporting our hypothesised demographic trade‐off with elevation. The earthquake altered growth–survival relationships from those found pre‐earthquake and 0–5 years post‐earthquake survival held little relationship with growth. A strong, logarithmic growth–survival relationship developed 5+ years post‐earthquake because of enhanced survival of fast‐growing trees yet low survival of slow‐growing trees. *Synthesis*. Our findings demonstrate a trend in growth–survival relationships along an elevation gradient. If we assume a gradual climate warming is the equivalent of a forest stand shifting to a lower elevation, then data from our pre‐earthquake period suggest that tree growth–survival relationships at any elevation could adjust to faster growth and lower survival. We also show how these novel growth–survival relationships could be altered by periodic disturbance.

## Graphical abstract

Tree growth–survival relationships link two demographic processes important to forest ecosystems. Trends in growth–survival relationships were demonstrated along an elevation gradient. The nature of these relationships was altered by an earthquake.

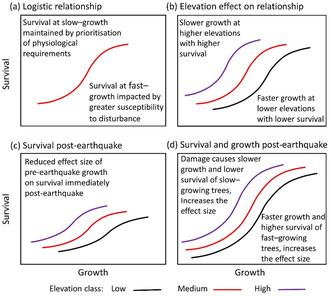

## 1 Introduction

**FIGURE 1:**
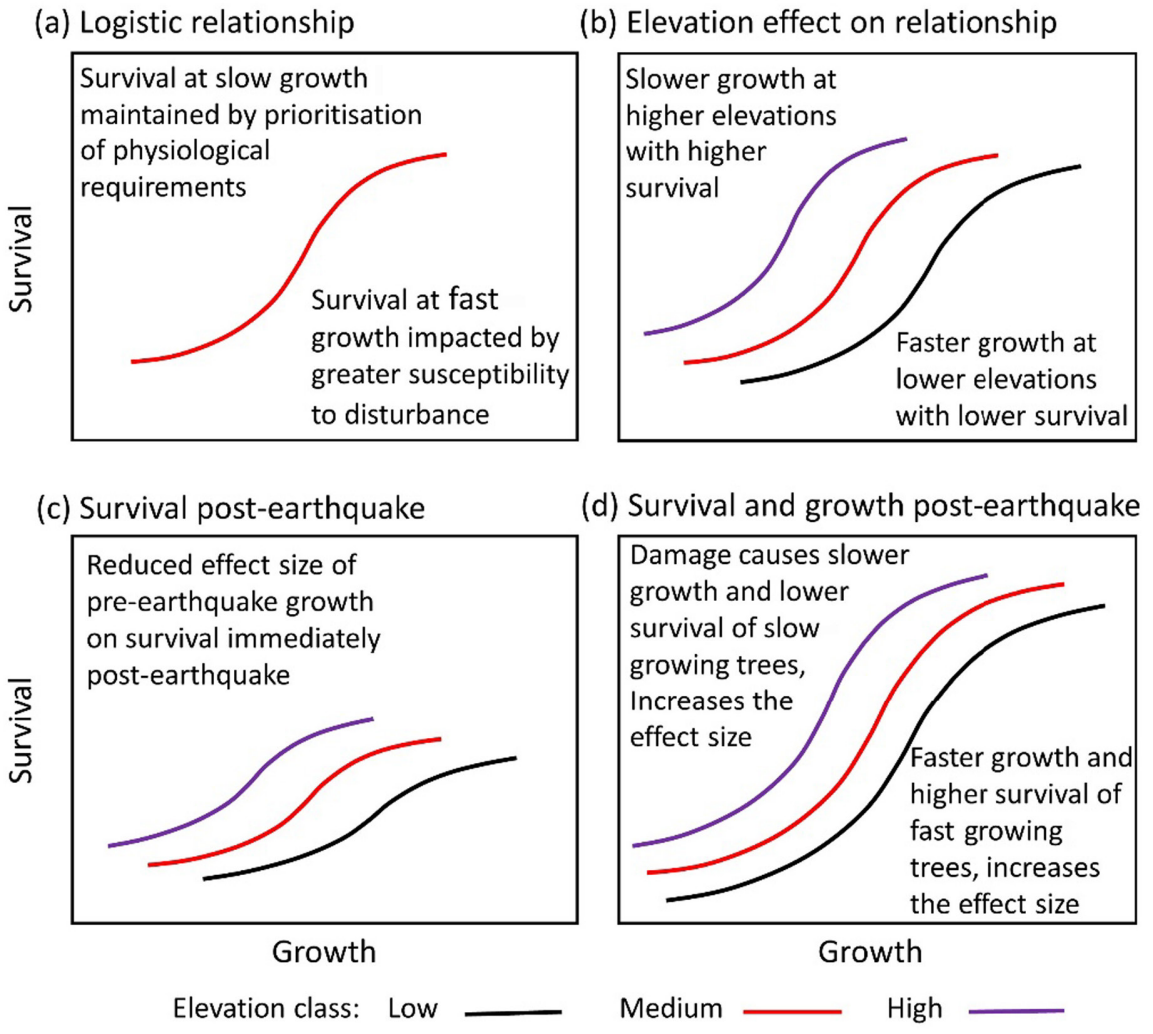
Hypothesised logistic relationships between tree growth rate (Growth) and survival probability (Survival) with slow‐ and fast‐growth tails where survival changes little with growth (a). Hypothesised progressive shift of the logistic growth–survival relationship to slower growth, but higher survival, with increasing elevation (b). Hypothesised reduction in survival at all elevations immediately post‐earthquake; tree survival is weakly related to growth creating a reduced effect size (c). Lower survival of damaged, slow‐growing trees post‐earthquake and faster growth and survival of fast‐growing trees because of competitive release were hypothesised to increase the effect size of the growth–survival relationship (d).

Tree mortality has long intrigued ecologists and foresters as it profoundly influences the composition, structure and functioning of forest ecosystems (Manion 1981; Pickett and White 1985; Mueller‐Dombois 1987; Franklin et al. 2002; Anderegg et al. 2015). Mortality patterns range from individual deaths due to ongoing neighbourhood competition or pathogen attack to extensive, synchronous death from infrequent disturbance (Peet and Christensen 1987; Franklin, Shugart, and Harmon 1987). It is often assumed that within a forest stand faster growing trees face lower mortality risks and that this can be revealed by examining how tree mortality depends on growth (e.g., Wunder et al. 2008; Macalady and Bugmann 2014; Cailleret et al. 2017; Russo et al. 2021). Recent changes (< 10 years) in tree diameter are often used to depict growth in such relationships (e.g., Wyckoff and Clark 2002; Cailleret et al. 2017). Hartmann et al. (2018) proposed a logistic relationship between a risk factor and tree mortality, and this conceptual model translates into a logistic relationship between tree growth and survival. This generic shape of the growth–survival relationship would have slow‐ and fast‐growing tails where survival changes little with growth (Figure 1a). The slow‐growth tail is plausible if the survival of individuals is maintained by prioritising physiological requirements, for example storage of carbon reserves, over growth (e.g., Huang et al. 2021; Wiley and Helliker 2012) or where the resources allocated to the growth of certain structures (e.g., roots or branches) are prioritised over diameter growth (e.g., Poorter et al. 2012). The fast‐growing tail if larger trees become more susceptible to disturbance (Figure 1a; Coomes et al. 2003; Wiley and Helliker 2012; Huang et al. 2021). This conceptual tree growth–survival relationship is consistent with empirical data for some tree species (e.g., Macalady and Bugmann 2014; Camac et al. 2018; Zuleta et al. 2022).

Empirically derived tree growth–survival relationships have been shown to vary intra‐specifically among locations. It remains unclear whether this reflects environmental variation (Wunder et al. 2008; Das, Stephenson, and Davis 2016; Camac et al. 2018). Elevation gradients provide a ‘natural experiment’ for examining how tree growth–survival relationships vary with environment (Körner 2007; Mayor et al. 2017). Elevation is a complex gradient where lower air and soil temperatures, shorter growing seasons, decreased nutrient supply and increased exposure often occur at higher elevations. These factors limit forest productivity at higher elevations and trees often grow slower (e.g., Paulsen, Weber, and Körner 2000; Coomes and Allen 2007a). If a single growth–survival relationship applied at all elevations, this slower growth would result in lower survival with increasing elevation. However, slower growth of neighbours at higher elevations can weaken the negative effects of competition (Callaway 1998; Coomes and Allen 2007a; Luo and Chen 2015; Das, Stephenson, and Davis 2016), potentially increasing survival (Stephenson et al. 2011). Therefore, we expected that tree growth–survival relationships would progressively shift to slower growth, but higher survival, with increasing elevation (Figure 1b; Macalady and Bugmann 2014; Camac et al. 2018).

The influence of elevation on tree growth–survival relationships may in turn be altered and weakened by disturbance (Russo et al. 2021). Earthquakes are a disturbance affecting forests throughout the world's tectonically active regions (e.g., Garwood, Janos, and Brokaw 1979; Galli et al. 2014; Kang et al. 2022). In montane regions, they cause landslides that remove whole forest stands on steep slopes (Allen, Bellingham, and Wiser 1999) and also generate rock falls that kill or injure individual trees (Keefer 1984). One view is that the stresses imposed by a disturbance would reduce the survival of trees that had been slow‐growing and thus potentially increase the effect size of growth–survival relationships (Manion 1981; Franklin, Shugart, and Harmon 1987). However, earthquake damage will kill some trees irrespective of their pre‐earthquake growth, and other trees will continue to die from the influence of neighbourhood competition (Allen, Bellingham, and Wiser 1999; Veblen et al. 2016; Kang et al. 2022). As a consequence, we expect a positive growth–survival relationship would persist immediately after an earthquake, but with a reduced effect size of pre‐earthquake growth on post‐earthquake survival (Figure 1c). Growth–survival relationships may also differ when post‐earthquake growth is related to post‐earthquake survival because earthquake damage may further reduce the survival of slow‐growing trees due to, for example, a build‐up of pathogens (Rawlings 1953). This, combined with a potential competitive release of some fast‐growing trees, might allow the growth–survival relationships to span a wider range of growth rates and have an increased effect size post‐earthquake (Figure 1d).

**FIGURE 2:**
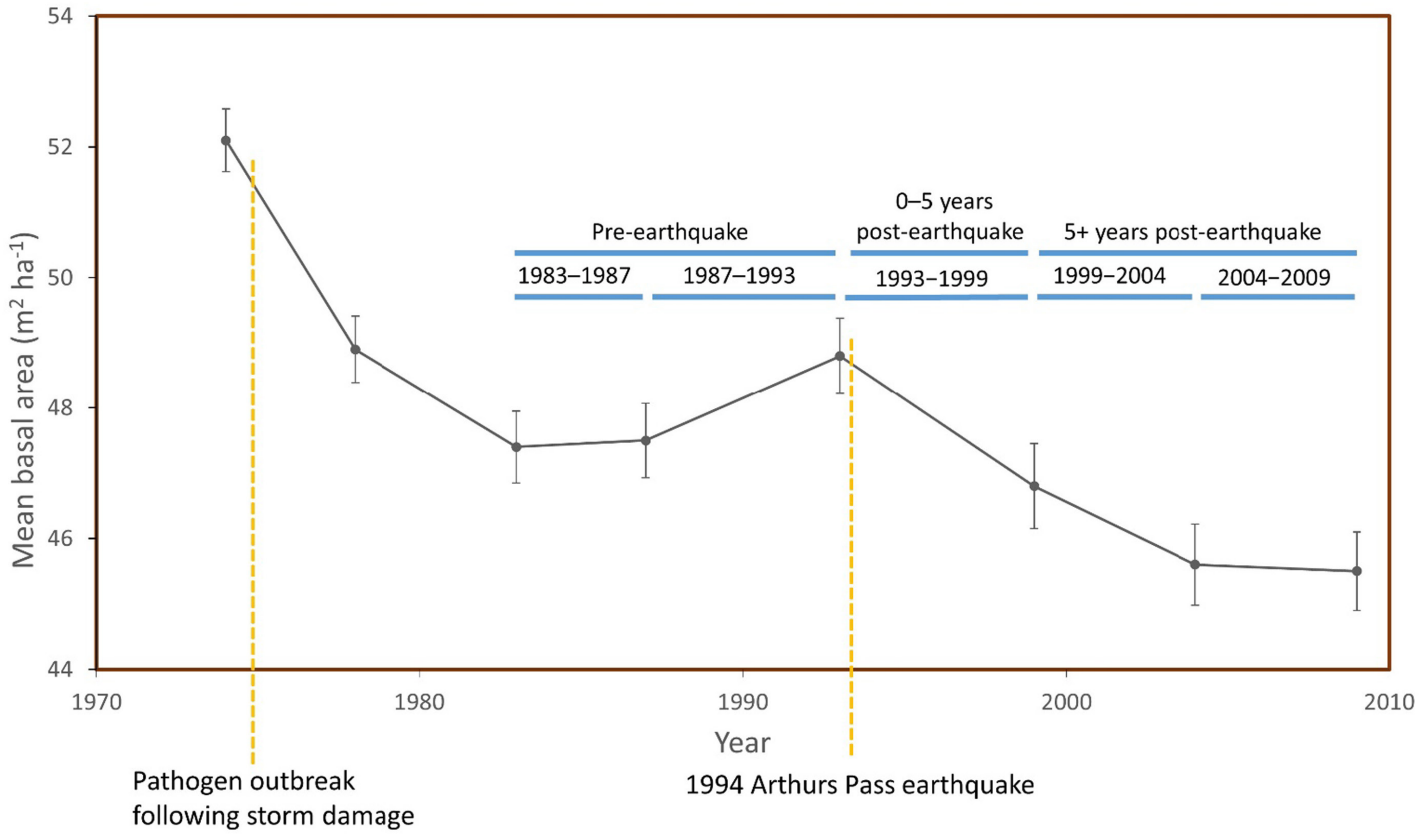
Mean (±SE) basal area (m^2^ ha^−1^) of mountain beech forest based upon measurements of 216 0.04 ha permanent plots over 35 years between 1974 and 2009 in the study area. Growth was determined in time windows preceding survival intervals. The five survival intervals used in our analyses fell within three survival periods: pre‐earthquake (with two intervals 1983–1987 and 1987–1993), 0–5 years post‐earthquake (a single interval 1993–1999) and 5+ years post‐earthquake (two intervals 1999–2004 and 2004–2009). The timing of the 1994 Arthurs Pass earthquake and a pathogen outbreak following storm damage are also indicated.

In this study, we examined variability in the shape of mountain beech (*Nothofagus solandri* var. *cliffortioides*) tree growth–survival relationships over 35 years (1974–2009) along an elevation gradient, both pre‐ and post‐earthquake. Understanding these relationships builds upon our previous research that determined what factors influence individual mountain beech tree survival or growth (Coomes and Allen 2007a, 2007b; Hurst et al. 2011; Allen et al. 2020). We used tagged trees on 216 plots that representatively sampled 7000 ha of old‐growth mountain beech forest (Allen et al. 2020). This angiosperm species is a long‐lived (250–350 years) evergreen tree that dominates montane forests in eastern parts of New Zealand (Wiser et al. 2011). We hypothesised that individual mountain beech tree survival models incorporating recent growth would be better supported than those only based upon other factors known to influence survival. Pre‐earthquake (1983–1993), when neighbourhood competition was relatively important to tree survival (Allen et al. 2020), we hypothesised that growth would relate logistically, and positively, to survival (Figure 1a; Macalady and Bugmann 2014; Camac et al. 2018). Although mountain beech tree growth declines with elevation (Coomes and Allen 2007a), we hypothesised that pre‐earthquake survival would increase with elevation because of reduced neighbourhood competition (Figure 1b; Coomes and Allen 2007a; Allen et al. 2020). This demographic trade‐off equates to a slower pace‐of‐life upslope (sensu Réale et al. 2010). The 1994 Arthurs Pass earthquake (*Mw* = 6.7) extensively damaged our forest (Figure 2; Hurst et al. 2011; Xu, Allen, and Newman 2023). We hypothesised that the effect of pre‐earthquake growth on immediate post‐earthquake survival (1993–1999) would be lower than that on pre‐earthquake survival (Figure 1b vs. Figure 1c). When we examined growth–survival relationships when both growth and survival were determined post‐earthquake (1999–2009), we hypothesised there would be lower survival of slow‐growing trees because of damage, faster growth and survival of some fast‐growing trees because of competitive release, and an increased effect size of the growth–survival relationship (Figure 1d). Finally, we use our observations to infer how growth–survival relationships may be altered in a warming world. Increasing temperatures with decreasing elevation provides a useful surrogate for warming.

## 2 Material and Methods

### 2.1 Study Area

The study was located in the forests of the Avoca and Harper Rivers which form part of the mountainous headwaters of the Rakaia River, South Island, New Zealand (Appendix S1: Figure S1). Mountain beech dominates the forests (> 99% of tagged trees) between the valley bottoms at c. 650 m and treeline at c. 1350 m elevation in the study area (Wiser et al. 2011). The terrain has been glaciated repeatedly and contains extensive morainal, fluvioglacial and periglacial slope deposits (Mosley 1979). Lower slopes and valley bottoms have extensive alluvial and colluvial deposits (Appendix S1: Figure S2).

Mean annual precipitation decreases from 2500 mm on the western edge of the study area to 1500 mm on the eastern edge, where mean annual temperature is 8.0°C at 914 m elevation (McCracken 1980). Mean annual precipitation increases by 30 mm per 100 m of elevation, and mean annual temperature has a lapse rate of 0.66°C per 100 m of elevation (McCracken 1980). Over the last five decades, variability in annual or growing season temperatures have been small when compared with that along the elevation gradient (Allen et al. 2014). Mountain beech stands decline in height, biomass and productivity, but increase in basal area, with increasing elevation (e.g., Benecke and Nordmeyer 1982; Harcombe et al. 1998; Coomes and Allen 2007a). Neighbourhood competition has a greater negative effect on tree growth and survival at low elevation, particularly for small trees (Coomes and Allen 2007a).

Damage from a heavy snowfall in 1973, and a subsequent build‐up of pathogens, led to dispersed canopy mortality and reduced study area basal area between 1974 and 1983 (Figure 2). Recruitment and stand development have subsequently led to basal area recovery between 1983 and 1993 (our pre‐earthquake survival period). During this period, tree growth and survival were strongly driven by neighbourhood competition and size (Coomes and Allen 2007a; Allen et al. 2020). The 1994 earthquake was centred c. 6 km north of the study area (Appendix S1: Figure S1; Arnadottir, Beavan, and Pearson 1995) and 11% of the plots near the epicentre (distance < 12 km) had > 80% basal area mortality, particularly on downslope landforms at lower elevations (Allen, Bellingham, and Wiser 1999; Appendix S1: Figure S2). Basal area declined in our first post‐earthquake survival period (1993–1999; Figure 2) and survival was less influenced by neighbourhood competition (Allen et al. 2020). Earthquake‐induced tree injury also impacted survival between 1999 and 2004 as basal area continued to decline (Allen, Bellingham, and Wiser 1999; Figure 2). By 2009, basal area had stabilised suggesting a level of recovery towards the end of our final post‐earthquake survival period (1999–2009; Figure 2).

### 2.2 Data Collation

The 216 plots were located systematically along 85 random transect lines (see Allen et al. 2020; Appendix S1: Figure S1). Transect lines had 1 to 8 plots. Each 0.04 ha (20 m × 20 m) plot was subdivided into 5 m × 5 m subplots (16 per plot) using tapes between opposing boundaries. Within each plot, the 1974 diameter at 1.4 m height was measured for each individual tree ≥ 30 mm in size and recorded by species, plot and subplot. Re‐measurements of the tagged trees were used to determine annual survival probability (hereafter survival) and annual diameter growth rate (hereafter growth in mm year^−1^). Recent changes (< 10 years) in tree diameter are widely used to generate individual growth metrics in ecological studies (e.g., Clark and Clark 1999; Wunder et al. 2008; Cailleret et al. 2017). Our growth metrics, based upon changes in diameter, reflect individual vitality and productivity (Dobbertin 2005; Cailleret et al. 2017). We use data with re‐measurements from the austral summers starting in 1978, 1983, 1987, 1993, 1999, 2004 and 2009.

We related growth to survival in three periods: when survival and growth were determined pre‐earthquake; when survival, but not growth, was post‐earthquake (0–5 years post‐earthquake); and, when survival and growth were both post‐earthquake (5+ years post‐earthquake). Within these periods, we estimated survival in five intervals (Figure 2): pre‐earthquake with two intervals 1983–1987 and 1987–1993; 0–5 years post‐earthquake a single interval 1993–1999; and 5+ years post‐earthquake, with two intervals 1999–2004 and 2004–2009. Two growth metrics were calculated for each survival interval: growth in a time window preceding death (previous growth); and growth in a time window preceding previous growth (lagged growth). For example, the growth covariates for the 1983–1987 survival interval were as follows: 1974–1978 for lagged growth; and, 1978–1983 for previous growth. Using data from c. 10 years preceding death covers a time window found significant for trees elsewhere (e.g., Table 1; Macalady and Bugmann 2014; Cailleret et al. 2017; Rodríguez‐Catón et al. 2019).

**TABLE 1:** Standardised scale used for each tree‐ and plot‐level covariate employed to model survival, and corresponding units for interpretation of estimated effect size as well as a covariate description.

Covariate	Zero on standardised scale	Units	Description
Tree‐level			
$\mathrm{ba}s_{i,t-1}$	50	m^2^ ha^−1^	Basal area
$y_{i,t-1}$	164	mm	Diameter at breast height
$G_{i,t-2}$		mm year^−1^	Previous growth
$G_{i,t-3}$		mm year^−1^	Lagged growth
Plot‐level			
$\mathrm{av}P_{j}$	exp(2.7) = 14.88	μg g^−1^	Soil‐available P
$LI_{j}$	20	Degrees	Landform index
$\mathrm{dis}t_{j}$	exp(2.9) = 18.17	ln(km)	Distance from the epicentre
$\mathrm{el}e_{j}$	1000	m	Elevation

**FIGURE 3:**
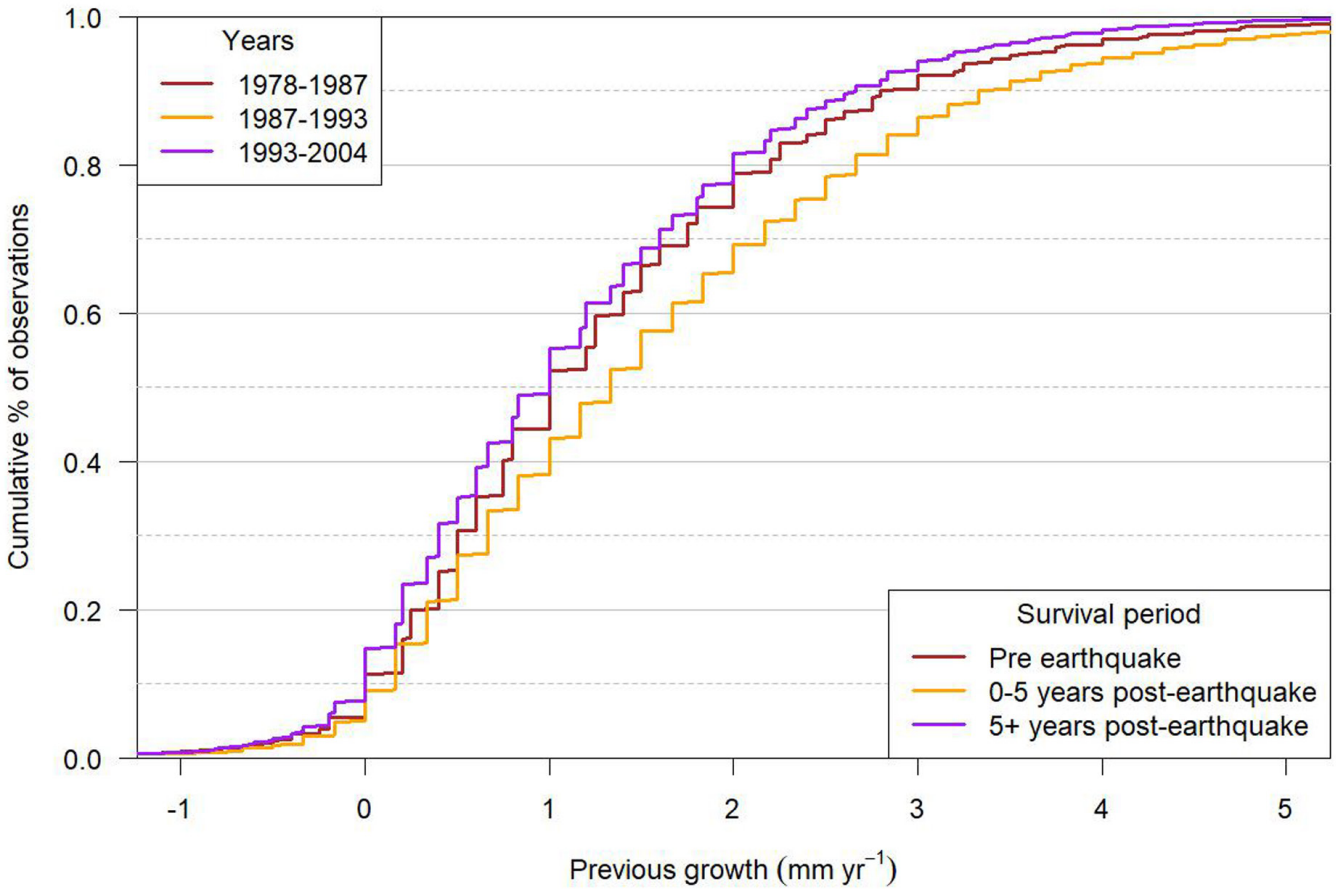
Cumulative % of observations versus previous growth (mm year^−1^). Previous growth is given for three time windows that were used to model survival within each of the three survival periods (pre‐earthquake, 0–5 years post‐earthquake and 5+ years post‐earthquake).

We accounted for two types of errors in our growth metrics. Rare outliers caused by extreme errors in diameter measurement or recording can skew growth estimates (Condit et al. 1999). To overcome these errors, we imputed diameter values using a modified version of an existing growth model (Appendix S2; Allen et al. 2020) for eight trees (0.2% of total trees) where growth was > 10 mm year^−1^ or < −3 mm year^−1^ (see Richardson et al. 2011) and nine trees (0.2% of total trees) where diameters were missing for a measurement. We retained trees in our models with small negative growths because these could be due to routine measurement error (Figure 3); excluding these would bias growth upwards in this slow‐growing species (Holdaway et al. 2014; Kenfack et al. 2014). While lifetime tree growth must be positive, stress (e.g., drought‐induced) and damage (e.g., bark shedding) can lead to diameter decreases and what appears to be short‐term negative growth (e.g., Clark and Clark 1999; Martínez‐Pastur et al. 2007).

To understand how tree growth influences tree survival we incorporated the effects of other tree‐ and plot‐level covariates that have previously been shown to influence the survival of mountain beech trees in the study area (Table 1; Coomes and Allen 2007b; Hurst et al. 2011; Allen et al. 2020). Neighbourhood competition was one of these covariates and for this we calculated for each tree, at each measurement, a local basal area (m^2^ ha^−1^) of all trees within a 15 m × 15 m square centred on the 5 m × 5 m subplot within which the tree was recorded. Coomes and Allen (2007a) showed that local basal area calculated within such a 15 m × 15 m square had greater explanatory power when predicting individual tree diameter growth than local basal area calculated within the 5 m × 5 m subplot a tree was located or the whole 20 m × 20 m plot. We also explicitly accounted for neighbourhood competition because it is affected by disturbance and recovery in the forests (e.g., Cavin et al. 2013). Diameter was also used as a tree‐level covariate. Using the geographic co‐ordinates of the earthquake epicentre and GPS locations for each plot, we calculated a distance (km) from the epicentre. Slope (degrees) and elevation (m) were determined as plot‐level covariates in 1974, and in 1993, we determined a landform index where the lowest values represented exposed ridge crests and the highest values sheltered downslope positions (McNab 1993). In 1992, mineral soil cores systematically collected from each plot were analysed for Bray 2 exchangeable P (μg g^−1^) as a measure of soil‐available P (see Allen et al. 2020). The tree‐ and plot‐level covariate values well represent their respective ranges (Figure 3; Appendix S1: Figure S3).

### 2.3 Statistical Methods

To examine intra‐specific variability in tree growth–survival relationships, we modelled survival not only as an effect of growth but also its combination with other tree‐ and plot‐level covariates. Our survival models employed a total of 4504 trees across measurements taken within the four central 5 × 5 m subplots of the 216 plots. Growth metrics, local basal area and diameter as covariates had tree‐level values used in the modelling for each measurement while the plot‐level covariates were constant over measurements.

Within our survival models, each covariate was associated with a coefficient that determines the direction, and strength (i.e., the effect size), of a relationship with tree survival. Our analysis allowed the value of model coefficients to vary over time, in accordance with the three defined survival periods. The pre‐earthquake and 5+ years post‐earthquake periods have one coefficient value in each survival model, although each has two time intervals that contribute (Figure 2). A change in model coefficient values among periods reflects changes in the nature of the relationship between covariates and survival. So that, for example, the sign of a model coefficient can be used to assess whether survival increased or decreased with elevation for any period. If the magnitude of a model coefficient is greater in one period than another, then it represents a greater effect size. Variability in growth–survival relationships among elevations was used to evaluate demographic trade‐offs over the full range of survivals and growths (cf. Russo et al. 2021).

Bayesian methods of inference estimated coefficients for model parameters, using the software JAGS from within the R statistical computing environment. Three Markov Chain Monte Carlo (MCMC) chains were run for 150,000 iterations following a burn‐in period of 10,000, with every third value being retained. Therefore, inferences were based upon posterior distributions approximated from 150,000 samples.

### 2.4 Selection of a Survival Model

A range of different survival models were fit to the data to compare the support for various combinations of the two potential growth covariates, along with all other standardised tree‐ and plot‐level covariates (Tables 1 and 2). The ‘full’ model (described below) included all covariates listed in Table 1 as well as the interactions of previous growth, or lagged growth, with elevation. Simpler survival models were considered by removing one or both growth covariates, or their interaction with elevation (Table 2). This allowed us to determine which growth covariate, and combination of growth covariates, had the greatest support. Note that the respective growth covariate was always retained in the model if the corresponding interaction with elevation was included. A ‘base’ model included all the tree‐ and plot‐level covariates but not any growth covariates (Table 2). Including a base model allowed us to determine if the growth covariates improved support above and beyond that provided by other tree‐ and plot level covariates. We expect that survival models with growth covariates would have more support than the base model if growth captures the influence of additional factors (e.g., tree damage or pathogen attack).

**TABLE 2:** Comparisons of alternative survival models fitted to the tagged tree data collected over 35 years, ranked in order of Watanabe–Akaike information criterion (WAIC). Given is the posterior mean of the model deviance, the WAIC penalty and relative difference in WAIC (ΔWAIC) between the top‐ranked and other models. Various combinations of two tree diameter growth covariates were used in models: Annual growth in a previous time window (mm year^−1^; previous growth) and annual growth in a time window lagged from previous growth (mm year^−1^, lagged growth). A base model (Base) included various tree‐ and plot‐level covariates, but without including any tree diameter growth covariates.

Model description	Mean deviance	WAIC penalty	ΔWAIC
Base + previous growth + lagged growth + previous growth × elevation interaction + lagged growth × elevation interaction	135,725.93	5320.62	0.00
Base + previous growth + lagged growth + previous growth × elevation interaction	135,730.53	5316.85	0.82
Base + previous growth + lagged growth + lagged growth × elevation interaction	135,744.05	5310.06	7.56
Base + previous growth + lagged growth	135,752.31	5305.00	10.75
Base + previous growth + previous growth × elevation interaction	135,815.99	5302.42	71.86
Base + previous growth	135,833.00	5294.49	80.93
Base + lagged growth + lagged growth × elevation interaction	136,015.56	5299.53	268.54
Base + lagged growth	136,026.05	5298.74	278.23
Base	136,202.93	5291.24	447.62

Models were compared using the Watanabe–Akaike information criterion (WAIC), a measure of the predictive performance, or support, for a model. WAIC was preferred over the Deviance information criterion (DIC) due to its greater reliability in certain circumstances (Gelman, Hwang, and Vehtari 2014). Note there are also multiple published definitions for DIC with different performance characteristics (e.g., Celeux et al. 2006; Plummer 2008). The deviance statistic calculated as $-2\times \ln\left(\mathrm{pr}\left(\text{data}|\theta \right)\right)$, where pr(data∣θ) is the probability of the data according to the defined model, given the values of the set of parameters in the model θ. A posterior distribution for the deviance statistic was obtained by evaluating it for each MCMC chain iteration.

### 2.5 Full Survival Model

Tree survival can be inferred from a binary sequence of observations of whether a tree is alive ($a_{i,t}=1$) or dead ($a_{i,t}=0$) during each measurement. Consecutive 1 s indicates the tree has survived between two periods, while a 1 followed by a 0 would indicate death.

Binary data can be modelled using the Bernoulli distribution, with an associated probability of the observed variable being 1. In this setting, let $S_{i,t-1}$ be the annualised probability of a tree surviving from time t−1 to *t*, given the tree was alive at time t−1 (i.e., $a_{i,t-1}=1$; $S_{i,t-1}=0$ otherwise). Therefore, the probability of a tree surviving the interval was $S_{i,t-1}^{\Delta t}$.

Survival probability was modelled on the logit scale, to allow incorporation of covariates in the same manner as logistic regression. Individual tree survival was modelled with a combination of tree‐ and plot‐level effects.

$$\begin{array}{c} \text{logit}\left(S_{i,t-1}\right)=\mu_{S,\mathrm{eq}}+\gamma_{y,\mathrm{eq}}y_{i,t-1}+\gamma_{y^{2},\mathrm{eq}}y_{i,t-1}^{2}\\ +\gamma_{\mathrm{bas},\mathrm{eq}}\mathrm{ba}s_{i,t-1}+\gamma_{\mathrm{bas}\times \mathrm{ele},\mathrm{eq}}\mathrm{ba}s_{i,t-1}\times \mathrm{el}e_{j}\\ +\gamma_{G,\mathrm{eq}}G_{i,t-2}+\gamma_{G\times \mathrm{ele},\mathrm{eq}}G_{i,t-2}\times \mathrm{el}e_{j}+\gamma_{G1,\mathrm{eq}}G_{i,t-3}\\ +\gamma_{G1\times \mathrm{ele},\mathrm{eq}}G_{i,t-3}\times \mathrm{el}e_{j}+P_{S,j,t-1} \end{array}$$

**FIGURE 4:**
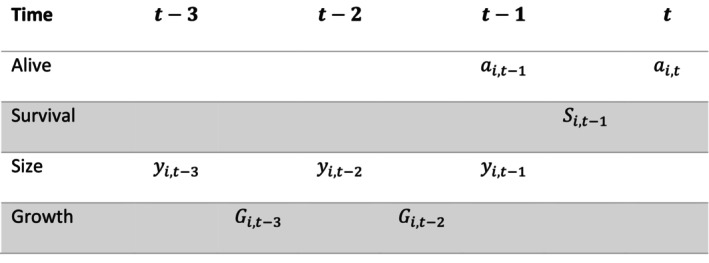
Schematic of time indexing for survival and growth. Note size (diameter size) and alive (otherwise dead) are recorded at the times of measurement whereas survival (alive at consecutive times) and growth occur between measurements.

Tree‐level effects included a tree diameter (*y*), a quadratic effect of tree diameter (*y* ^2^), basal area (bas), an interaction between basal area and elevation (ele), annual growth in two time windows (G−previous growth at *t*−2; G1−lagged growth at *t*−3) before a survival interval (all as separate covariates; Figure 4), and also interactions between growth and elevation, in earthquake survival period eq. Incorporating survival period in the model allowed us to present the results and model inferences for the pre‐earthquake, 0–5 years post‐earthquake and 5+ years post‐earthquake periods separately. Annual growth was calculated for each time window as:

$$G_{i,t}=\frac{y_{t}-y_{t-1}}{\Delta t}$$

Plot‐level effects ($P_{S,j,t-1}$) were modelled as functions of the plot‐level covariates distance from the epicentre (dist), soil‐available P (avP), landform index (LI) and elevation (ele), and a random plot‐level effect that was assumed to be normally distributed with mean = 0 and variance $\sigma_{S}^{2}$. Covariate coefficients for the survival model parameters are denoted as $\gamma_{X,\mathrm{eq}}$ for covariate X, in earthquake survival period eq.

$$P_{S,j,t-1}=\gamma_{\text{dist},\mathrm{eq}}\mathrm{dis}t_{j}+\gamma_{\mathrm{avP},\mathrm{eq}}{\mathrm{avP}}_{j}+\gamma_{\mathrm{LI},\mathrm{eq}}LI_{j}+\gamma_{\mathrm{ele},\mathrm{eq}}\mathrm{el}e_{j}+\epsilon_{j}$$

In summary, the stochastic elements of the survival model were defined as:

$$a_{i,t}\;\text{Bern}\left(S_{i,t-1}\right),\;\text{if}\;a_{i,t-1}=1$$

$$\epsilon_{j}\;N\left(0,\sigma_{S}^{2}\right)$$

**FIGURE 5:**
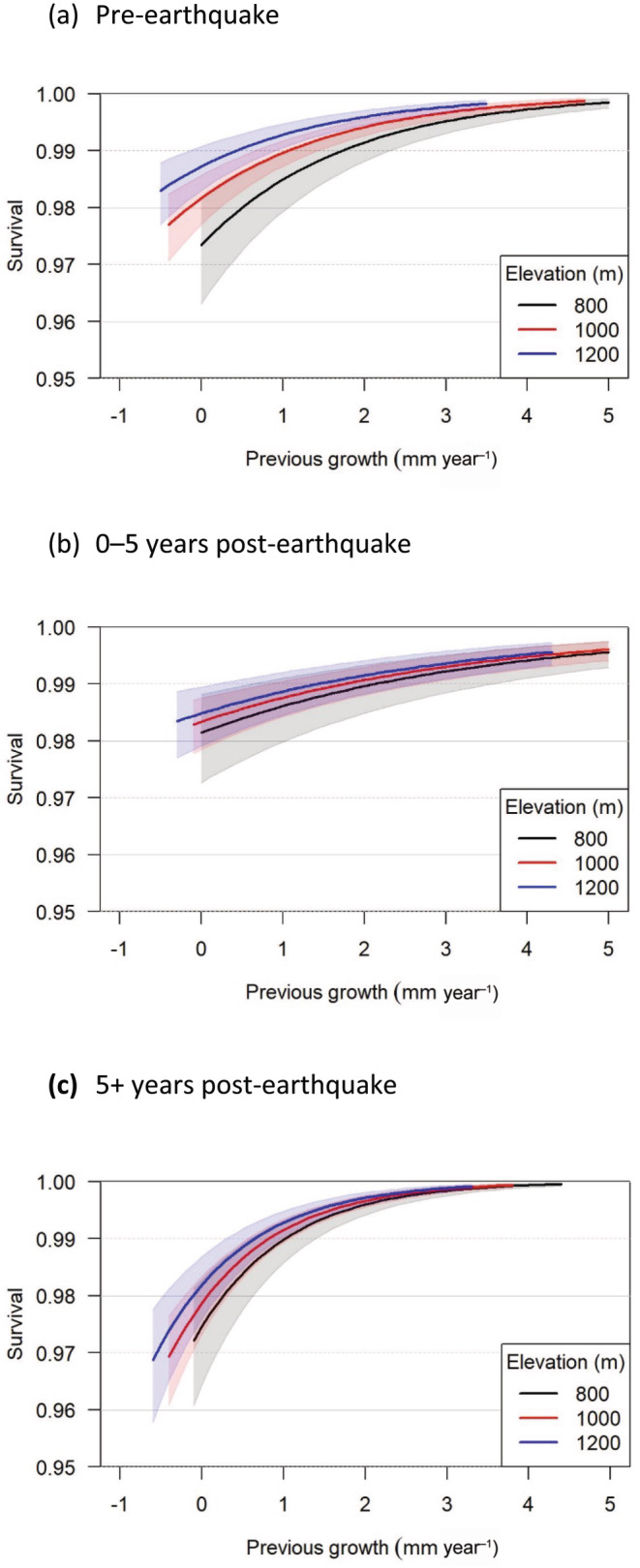
Mean annual survival probability (survival; excluding effect of random plot‐level variation) from the selected model in pre‐earthquake ((a); 1983–1993), 0–5 years post‐earthquake ((b); 1993–1999) and 5+ years post‐earthquake ((c); 1999–2009) periods plotted against previous growth (mm year^−1^), respectively. Previous growth was constrained to be within the central 95% of values for each period in three elevation classes: low = 640–970 m, medium = 971–1130 m and high = 1131–1417 m. The plots illustrate the effect of previous growth at 800, 1000 and 1200 m elevation, the median plot elevation in each of three classes, with standardised values of all other covariates used in the model set to zero. Because there was little correlation between covariates, the effect will be the same when the other standardised covariates are not zero, but the magnitude will be different. Shaded areas indicate 95% credible interval.

Coefficients given for parameters include those for the overall model where all covariates in the model are zero on the standardised scale (Table 3). Zero on the standardised scale has implications for understanding model output. A simple expectation might be lowered survival 0–5 years post‐earthquake, but this is not necessarily so at zero on the standardised scale for covariates (e.g., the mid‐point of distance from the earthquake epicentre; Table 1). Parameter coefficients are also given for each tree‐ and plot‐level covariate, where all other covariates are zero on the standardised scale (Table 1). Similarly, the graphical representation of the growth–survival relationship is where all other covariates are zero on the standardised scale (Figure 5).

**TABLE 3:** Summary of posterior distributions (PD) of parameters for the selected survival model (on logit scale) fitted to the tagged tree data collected over 35 years. Parameters include those for the overall model as well as those for tree‐ and plot‐level covariates. A description is given of each parameter followed by the mean, standard deviation (SD) and percentile values of model coefficients for the PD. Parameter values are for the point where other covariates in the model are zero on their standardised scales (Table 1). As there was little correlation between covariates, the effect will be the same when other standardised covariates are not zero, but the magnitude will be different.

Parameter	Description	Mean	SD	Percentile values	
				2.5%	97.5%
Overall					
$\mu_{S,1}$	Mean survival pre‐earthquake (logit scale)	3.691	0.128	3.444	3.946
$\mu_{S,2}$	Mean survival 0–5 years post‐earthquake (logit scale)	3.896	0.163	3.578	4.221
$\mu_{S,3}$	Mean survival 5+ years post‐earthquake (logit scale)	3.667	0.133	3.408	3.932
$\sigma_{S}$	SD of plot‐level random effect	0.966	0.061	0.851	1.089
Tree‐level					
$\gamma_{y,1}$	Pre‐earthquake linear effect of diameter	0.00485	0.00076	0.00336	0.00635
$\gamma_{y^{2},1}$	Pre‐earthquake quadratic effect of diameter	−0.00002	0.00000	−0.00003	−0.00002
$\gamma_{y,2}$	0–5 years post‐earthquake linear effect of diameter	0.00563	0.00099	0.00369	0.00761
$\gamma_{y^{2},2}$	0–5 years post‐earthquake quadratic effect of diameter	−0.00002	0.00000	−0.00003	−0.00001
$\gamma_{y,3}$	5+ years post‐earthquake linear effect of diameter	0.00201	0.00085	0.00036	0.00368
$\gamma_{y^{2},3}$	5+ years post‐earthquake quadratic effect of diameter	−0.00001	0.00000	−0.00002	−0.00001
$\gamma_{\mathrm{bas},1}$	Pre‐earthquake effect of basal area	−0.011	0.005	−0.021	−0.001
$\gamma_{\mathrm{bas},2}$	0–5 years post‐earthquake effect of basal area	−0.002	0.006	−0.014	0.009
$\gamma_{\mathrm{bas},3}$	5+ years post‐earthquake effect of basal area	0.002	0.005	−0.007	0.011
$\gamma_{\mathrm{bas}\times \mathrm{ele},1}$	How the pre‐earthquake effect of basal area changes with elevation	−0.004	0.002	−0.009	0.001
$\gamma_{\mathrm{bas}\times \mathrm{ele},2}$	How the 0–5 years post‐earthquake effect of basal area changes with elevation	0.009	0.003	0.003	0.015
$\gamma_{\mathrm{bas}\times \mathrm{ele},3}$	How the 5+ years post‐earthquake effect of basal area changes with elevation	−0.005	0.003	−0.010	0.000
$\gamma_{G,1}$	Pre‐earthquake effect of previous growth	0.575	0.066	0.447	0.704
$\gamma_{G,2}$	0–5 years post‐earthquake effect of previous growth	0.187	0.058	0.074	0.301
$\gamma_{G,3}$	5+ years post‐earthquake effect of previous growth	0.776	0.072	0.636	0.919
$\gamma_{G\times \mathrm{ele},1}$	How the pre‐earthquake effect of previous growth changes with elevation	−0.054	0.029	−0.112	0.004
$\gamma_{G\times \mathrm{ele},2}$	How the 0–5 years post‐earthquake effect of previous growth changes with elevation	0.062	0.032	−0.002	0.125
$\gamma_{G\times \mathrm{ele},3}$	How the 5+ years post‐earthquake effect of previous growth changes with elevation	0.123	0.037	0.051	0.195
$\gamma_{G1,1}$	Pre‐earthquake effect of lagged growth	0.361	0.056	0.252	0.471
$\gamma_{G1,2}$	0–5 years post‐earthquake effect of lagged growth	0.261	0.066	0.132	0.391
$\gamma_{G1,3}$	5+ years post‐earthquake effect of lagged growth	0.288	0.050	0.192	0.389
Plot‐level					
$\gamma_{\mathrm{avP},1}$	Pre‐earthquake effect of soil‐available P	−0.047	0.117	−0.278	0.182
$\gamma_{\mathrm{avP},2}$	0–5 years post‐earthquake effect of soil‐available P	−0.218	0.132	−0.476	0.039
$\gamma_{\mathrm{avP},3}$	5+ years post‐earthquake effect of soil‐available P	0.072	0.121	−0.167	0.309
$\gamma_{\mathrm{LI},1}$	Pre‐earthquake effect of landform index	−0.009	0.023	−0.054	0.036
$\gamma_{\mathrm{LI},2}$	0–5 years post‐earthquake effect of landform index	−0.092	0.025	−0.141	−0.043
$\gamma_{\mathrm{LI},3}$	5+ years post‐earthquake effect of landform index	−0.026	0.024	−0.074	0.022
$\gamma_{\mathrm{ele},1}$	Pre‐earthquake effect of elevation	0.242	0.065	0.114	0.371
$\gamma_{\mathrm{ele},2}$	0–5 years post‐earthquake effect of elevation	−0.006	0.082	−0.167	0.153
$\gamma_{\mathrm{ele},3}$	5+ years post‐earthquake effect of elevation	0.023	0.067	−0.108	0.153
$\gamma_{\text{dist},1}$	Pre‐earthquake effect of distance from epicentre	−0.055	0.237	−0.522	0.411
$\gamma_{\text{dist},2}$	0–5 years post‐earthquake effect of distance from epicentre	1.036	0.254	0.543	1.538
$\gamma_{\text{dist},3}$	5+ years post‐earthquake effect of distance from epicentre	−0.004	0.248	−0.491	0.478
Deviance		135,730.527	185.136	135,369.702	136,096.378

## 3 Results

### 3.1 Selection of a Survival Model

Tree survival models incorporating growth were better supported than those simply based upon other factors known to influence survival. The top‐ranked model fitted to the tagged tree data was the full model (based upon WAIC value), which included interactions between previous growth and elevation, as well as lagged growth and elevation (Table 2). The WAIC value for the second‐ranked model was different by only 0.82, indicating a similar level of support to the full model. The mean deviance of the top‐ and second‐ranked models also differed little suggesting a similar model fit (Table 2). Both included the base model tree‐and plot‐level covariates. The structure of the second‐ranked model was the same as the top‐ranked model, except the interaction between lagged growth and elevation was excluded (Table 2). Therefore, there is strong evidence that the effect of previous growth changed with elevation, but it is ambiguous whether the effect of lagged growth also changed with elevation. As parameter posterior distributions from both models were very similar, we based our inferences on the slightly simpler second‐ranked model (Table 3; Results for the top‐ranked model are given in Appendix S3: Table S1 for comparison).

There was much greater support for previous growth affecting survival (∆WAIC = 80.93) than lagged growth (∆WAIC = 278.23), although all survival models with both previous and lagged growth covariates as main effects had much more support than those with only one of these main effects. In the former, the relative differences in WAIC were small (≤ 10.75), whereas in the latter, they were large (∆WAIC ranged from 71.86 to 278.23; Table 2). Thus, both previous growth and lagged growth influenced survival.

### 3.2 Pre‐Earthquake Growth–Survival Relationship

The dependence of survival on previous growth appeared logarithmic rather than, as hypothesised, logistic (Figure 5a vs. Figure 1a). Pre‐earthquake mean tree survival between 1983 and 1993 was high and is evident in the posterior distribution (PD) for $\mu_{S,1}$ in Table 3. At the point where all the standardised covariates were zero, pre‐earthquake mean survival (i.e., excluding plot‐level variation) was 0.982 with a 95% credible interval (CrI) of 0.977–0.986. There was considerable plot‐level variation in survival after accounting for the effects of covariates ($\sigma_{S}$). The probability of survival increased with previous growth (Figure 5a; PD for $\gamma_{G,1}>0$), as zero lies outside the 95% CrI (Table 3). As expected, there was a negative relationship between neighbourhood competition ($\gamma_{\mathrm{bas},1}$; local basal area) and survival (Table 3), as well as previous growth (Appendix S2: Table S2). Similarly, survival increased with lagged growth ($\gamma_{G1,1}$), although the effect size was smaller than for previous growth (Table 3).

There was evidence for a demographic trade‐off with elevation. The growth–survival relationship spanned a lower range of previous growths at higher elevations than at lower elevations (Figure 5a) and pre‐earthquake growth declined with increasing elevation ($\beta_{\mathrm{ele},1}$ in Appendix S2: Table S2). There was also a strong positive effect of elevation on survival ($\gamma_{\mathrm{ele},1}$; Table 3). The 95% CrI for the interaction term between previous growth and elevation ($\gamma_{G\times \mathrm{ele},1}$) included zero and suggested that the pre‐earthquake effect of previous growth on survival changed little with elevation. This was consistent with little separation in the growth–survival relationship at different elevations for faster growing trees (> 2 mm year^−1^; Figure 5a). However, there was separation in the growth–survival relationship at different elevations for the 40% of trees that grew slowly (≤ 1 mm year^−1^), with lowest survival, for a given level of previous growth, at low elevations (Figures 3 and 5a). This created the positive effect of elevation on survival.

### 3.3 0–5 Years Post‐Earthquake Growth–Survival Relationship

As hypothesised, the effect size of previous growth, and to a lesser degree lagged growth, on survival was least in the 0–5 years post‐quake period ($\gamma_{G,2}$ and $\gamma_{G1,2}$ had the lowest PD means for respective periods; Table 3; Figure 1c). At the point where all standardised covariates were zero, 0–5 years post‐earthquake mean survival (one interval between 1993 and 1999) was 0.983 (95% CrI: 0.978–0.987). We expected 0–5 years post‐earthquake survival to be lower than pre‐earthquake survival, but this was not so at zero on the standardised scale for covariates (e.g., the mid‐point of distance from the earthquake epicentre). The probability of survival generally increased logarithmically with previous growth, and, to a lesser degree, for lagged growth.

In the 0–5 years post‐earthquake period, growth declined with increasing elevation ($\beta_{\mathrm{ele},2}$ in Appendix S2: Table S2) and, as for pre‐earthquake, the growth–survival relationship spanned a lower range of previous growths at higher elevations than lower elevations (Figure 5b). Even though growth declined with elevation, there was no evidence for a main effect of elevation on survival ($\gamma_{\mathrm{ele},2}$) (Table 3). The interaction terms between basal area and elevation indicated that a positive effect of basal area on survival increased with elevation during this period ($\gamma_{\mathrm{bas}\times \mathrm{ele},2}$). Survival was lower for fast‐growing trees at low elevations and for slow‐growing trees at high elevations when compared with that pre‐earthquake (Figure 5b vs. Figure 5a). On the other hand, survival was relatively high for slow‐growing trees at low elevations when compared with that pre‐earthquake (Figure 5b vs. Figure 5a).

### 3.4 5+ Years Post‐Earthquake Growth–Survival Relationship

As hypothesised, the effect size of previous growth on survival was greatest in the 5+ years post‐quake period ($\gamma_{G,3}$), when compared with the other two periods, but this was not true for lagged growth ($\gamma_{G1,3}$; Table 3; Figure 1d). At the point where all standardised covariates were zero, 5+ years post‐earthquake mean survival (two intervals between 1999 and 2009) was 0.979 (95% CrI: 0.973–0.983). The probability of survival increased strongly and logarithmically with previous growth, and, to a lesser degree, for lagged growth (Table 3).

5+ years post‐earthquake, growth declined with increasing elevation ($\beta_{\mathrm{ele},3}$ in Appendix S2: Table S2) and, as in the earlier periods, the growth–survival relationship spanned a lower range of previous growths at higher elevations than at lower elevations (Figure 5c). There was little evidence for a main effect of elevation on survival ($\gamma_{\mathrm{ele},3}$). The interaction term between previous growth and elevation indicated that the effect of previous growth on survival increased with elevation during this period ($\gamma_{G\times \mathrm{ele},3}$; Table 3). This elevational effect is represented by a small shift in the growth–survival relationship to higher survivals with increasing elevation when previous growth is > 1 mm year^−1^ (Figure 5c). Survival was particularly low for trees with low previous growth at higher elevations when compared with that in the other two periods (Figure 5). This survival period is the one where previous growth was post‐earthquake, and it was not surprising that the growth–survival relationship extended further into negative growths than during earlier periods (Figure 5).

## 4 Discussion

Our 35‐year time series showed marked intra‐specific variability in the tree growth–survival relationships in a monodominant forest.

### 4.1 Selection of a Survival Model

As hypothesised, survival models incorporating growth were strongly supported, particularly when using previous growth rather than lagged growth. This emphasised the sensitivity of mountain beech survival to growth in the previous 5 years (Table 2). Other angiosperm tree species show small, short‐term growth reductions (47%) before death when compared with gymnosperm tree species (59%; Cailleret et al. 2017). Cailleret et al. (2017) offer a range of plausible mechanisms for this including a greater ability of angiosperms to flexibly utilise and store non‐structural carbohydrates for growth. On the other hand, mountain beech is light demanding and such species often show less marked reductions in growth before death (Wardle 1984; Wyckoff and Clark 2002; Wunder et al. 2008).

### 4.2 Pre‐Earthquake Growth–Survival Relationships

There is little consensus on the shape of tree growth–survival relationships, although empirical studies often describe survival as being positively related to growth (e.g., Wyckoff and Clark 2002; Wunder et al. 2008; Camac et al. 2018; Rodríguez‐Catón et al. 2019). Our survival probability was modelled on a logit scale but this did not detect a hypothesised slow‐growing tail for this positive relationship (Figure 1a). Many studies do not present slow‐growing tails but, as we found, do present fast‐growth tails (e.g., Pacala et al. 1996; Wyckoff and Clark 2002). Such fast‐growth tails are plausible if the anticipated increase in survival of fast‐growing trees is tempered by reduced survival due to disturbance. Certainly, large mountain beech trees appear susceptible to mortality (Coomes et al. 2003; Hurst et al. 2011).

We found support for a demographic trade‐off that reflects a slower pace‐of‐life upslope (Figure 1b). Slower growth with increasing elevation was expected (Coomes and Allen 2007a) although this is not always so intra‐specifically (Monserud and Sterba 1996; Rapp et al. 2012). A common garden experiment, using mountain beech seedlings, showed slower growth at higher elevation may be genetically controlled (Wilcox and Ledgard 1983). There was also a progressive shift from a wide range of growths at low elevation to a narrow range of growths at high elevation (Figure 5a). A slower pace‐of‐life also occurred because of a progressive increase in the survival of slow‐growing trees with increasing elevation, although the survival of fast‐growing trees remained remarkably unchanged (Figure 5; Table 3). A plausible explanation for this elevational adjustment is that tree growth decreases as a physiological response to a cooling environment which in turn reduces neighbourhood competition and thus increasing survival of small trees in particular (Callaway 1998; Coomes and Allen 2007a; de Toledo et al. 2011; Das, Stephenson, and Davis 2016). The negative effects of neighbourhood competition are focussed on the small, shaded individuals, rather than the larger individuals, and this light competition is most apparent at low elevations (Coomes and Allen 2007a). Elsewhere, higher survival at high elevation has been attributed to decreasing importance of drought (Das et al. 2013). However, at low elevations in our study area mountain beech photosynthesis is rarely constrained by moisture deficits (Benecke and Nordmeyer 1982).

Our intra‐specific demographic trade‐off mirrors an inter‐specific trade‐off from faster growth and lower survival on productive sites shifting to slower growth and higher survival on unproductive sites (Russo et al. 2008; Courbaud, Vieilledent, and Kunstler 2012; Laiolo and Obeso 2017). For trees, this inter‐specific demographic trade‐off represents a shift from fast‐growing, resource‐acquiring species with low investment in leaf tissues on productive sites to slow‐growing, resource‐conserving species with greater investment in leaf tissue on unproductive sites (Grime 1977; Reich 2014). This parallels a decline in leaf nutrient concentrations, but an increase in mountain beech leaf thickness and leaf mass per unit area, with elevation (Richardson et al. 2013). Indeed, many species exhibit considerable intra‐specific leaf trait variation (Siefert et al. 2015; Umaña and Swenson 2019). This suite of traits related to a slower pace‐of‐life upslope may be why trees are often older near treeline (e.g., Allen and Peet 1990).

### 4.3 0–5 Years Post‐Earthquake Growth–Survival Relationships

The stress imposed by a disturbance could enhance the mortality of slow‐growing trees (Manion 1981; Franklin, Shugart, and Harmon 1987). This is sometimes so after fire and wind disturbance, as tree death can be the result of several tree‐level characteristics including slow growth (van Mantgem et al. 2003; Tanner et al. 2014; Nesmith et al. 2015). That disturbance would enhance the mortality of slow‐growing trees did not strongly apply in our mountain beech forest because, as hypothesised, the effects size of the 0–5 years post‐earthquake growth–survival relationship was the smallest of the three periods (Figure 5b; Table 3). This was likely due to the indiscriminate impacts of earthquake‐induced landslides on the survival of some trees (Allen et al. 2020). In our study area, 24% of trees across the full range of sizes were immediately killed near the earthquake epicentre, of which 70% were killed by various forms of landslides (Allen, Bellingham, and Wiser 1999).

The low survival of fast‐growing trees 0–5 years post‐earthquake, particularly for low elevations, when compared with pre‐earthquake, may be because landslides damage forests on downslope positions (Figure 5b; Allen et al. 2020). Our 0–5 years post‐earthquake analyses showed complexity in the nature of intra‐specific tree growth–survival relationships. In a similar way, Russo et al. (2021) suggested, based upon inter‐specific variability in growth–survival relationships, that simple growth–survival trade‐offs may not provide a general framework for forest dynamics because of disturbance.

### 4.4 5+ Years Post‐Earthquake Growth–Survival Relationships

In this period, both growth and survival had the imprint of earthquake damage. Twenty‐three percent of trees were injured in 1994 near the earthquake epicentre, with many being damaged by other trees and rock falls (41% and 17%, respectively; Allen, Bellingham, and Wiser 1999). In 1995, 78% of these injured trees remained alive. We suggest that such tree injury led to the greater cumulative frequency of relatively slow‐growing trees, with low survival, 5+ years post‐earthquake (Figures 3 and 5c). Potentially, stress (e.g., induced by root damage influencing water and nutrient uptake) and damage (e.g., rock falls removing bark and wood at diameter measurement height) caused reduced, and sometimes negative, changes in diameter (e.g., Martínez‐Pastur et al. 2007; García‐Cervigón, Camarero, and Espinosa 2017). This accentuated the slope of the growth–survival relationship and created, as hypothesised, the greatest effect size for growth–survival relationships in our the three periods (Figure 5; Table 3). The particularly low survival of slow‐growing trees 5+ years post‐earthquake at high elevations, when compared with that pre‐earthquake, may be because low temperatures and low nutrient availability combined with injury to restrict tree vigour and survival (Benecke and Nordmeyer 1982; Coomes and Allen 2007a). Kunstler et al. (2021) considered that a cold distributional limit would reduce growth but not survival. Our results agree with this in the period of relative stability pre‐earthquake but not after trees had been affected by an earthquake.

## 5 Forest Dynamics in a Warming World

Our growth–survival relationships suggest a subtle consequence of a changing climate on the functioning of forests. If we assume that any given elevation is subjected to a warming, then this is equivalent to a forest stand shifting to a lower elevation. We accept that elevation is a complex gradient, but consider that many related factors, such as litter decomposition and neighbourhood competition, would similarly vary with a changing climate. In our pre‐earthquake period, at any given elevation, tree growth–survival relationships would adjust to faster growth and lower survival, particularly of slow‐growing trees (cf. Searle and Han 2018). This adjustment would reflect both abiotic (e.g., climate, resource availability) and biotic (neighbourhood competition) processes, as well as their interactions. We suspect that an increased pace‐of‐life would ensue at large scales. However, at the small scale (7000 ha) of our study, when the forest was disturbed by an earthquake, any demographic growth–survival trade‐offs with elevation were altered by disturbance. This alteration may be because disturbance influences the competitive environment and resource availability, but we also suggest there were strong legacies through the direct impact on individuals (Franklin et al. 2002).

## Author Contributions

**Robert B. Allen:** conceptualization (lead), data curation (lead), formal analysis (supporting), funding acquisition (lead), investigation (lead), methodology (lead), project administration (lead), resources (lead), supervision (lead), writing – original draft (lead), writing – review and editing (lead). **Darryl I. MacKenzie:** conceptualization (supporting), formal analysis (lead), methodology (lead), resources (supporting), software (lead), visualization (lead), writing – original draft (supporting), writing – review and editing (supporting). **Susan K. Wiser:** conceptualization (supporting), data curation (lead), formal analysis (supporting), investigation (supporting), methodology (supporting), resources (lead), visualization (supporting), writing – original draft (supporting), writing – review and editing (supporting). **Peter J. Bellingham:** conceptualization (supporting), data curation (supporting), funding acquisition (supporting), methodology (supporting), visualization (supporting), writing – original draft (supporting), writing – review and editing (supporting). **Lawrence E. Burrows:** conceptualization (supporting), data curation (supporting), methodology (supporting), visualization (supporting), writing – original draft (supporting), writing – review and editing (supporting). **David A. Coomes:** conceptualization (supporting), investigation (supporting), methodology (supporting), visualization (supporting), writing – original draft (supporting), writing – review and editing (supporting).

## Conflicts of Interest

The authors declare no conflicts of interest.

## Supporting information


Appendix S1

## Data Availability

The data are archived in, and can be requested from, New Zealand's National Vegetation Survey databank (https://nvs.landcareresearch.co.nz/).
