# Supplementary material for: A Growth–Survival Trade‐Off Along an Elevation Gradient Is Altered by Earthquake Disturbance in a Monodominant Southern Beech Forest
Source: Ecol Evol. 2024 Oct 31;14(11):e70467. doi: 10.1002/ece3.70467 (PMC11525070; doi:10.1002/ece3.70467)
Supplement: Supplementary file 1 — Appendix S1 [file ECE3-14-e70467-s001.docx]

**Appendix S1.** Map of the study area.


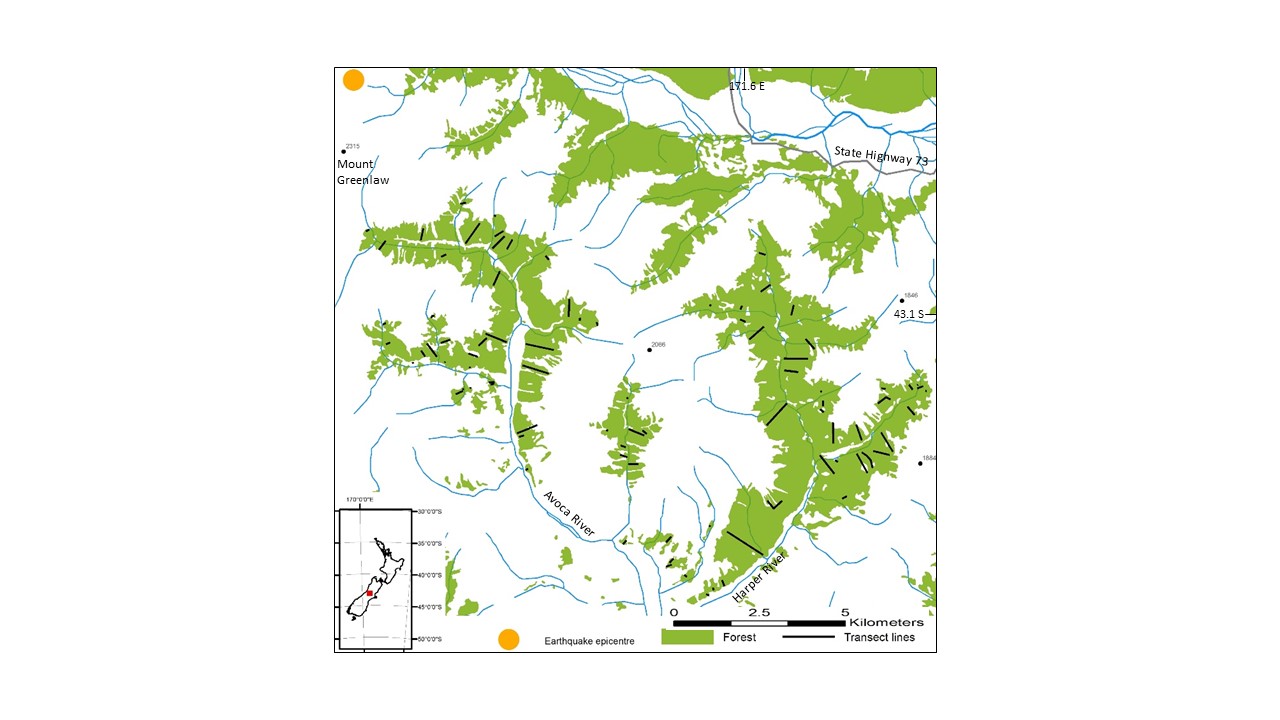


**Fig. S1.** Map of the study area showing location in New Zealand, latitude, longitude, earthquake epicentre, rivers, as well as transect lines in the mountain beech forest study area. The location of 85 transect lines (containing a total of 216 plots) sampling these forests, and their direction, is indicated using black lines. Elevation (m) of high points along the mountain range ridges are also given.


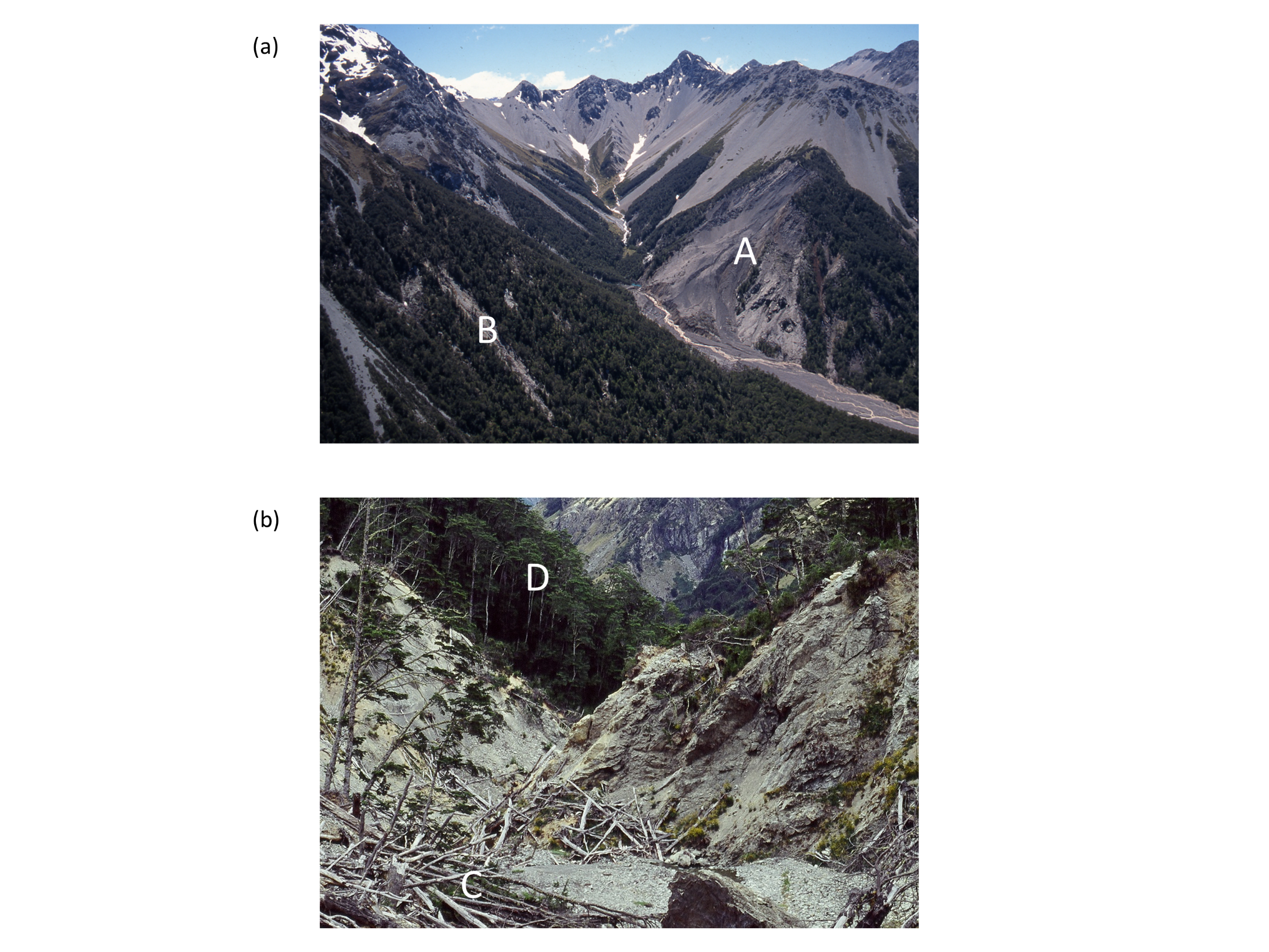


**Fig. S2.** Large earthquake-induced landslide that denuded forested slopes over a large area (A) and with lesser landslides in the foreground (B) causing smaller-scale disturbance, head of the Avoca River, approximately 7 km from the epicentre (photograph taken by John Barran in February 1995).

**Tree–level**

1. **Basal area (b) Diameter**

**
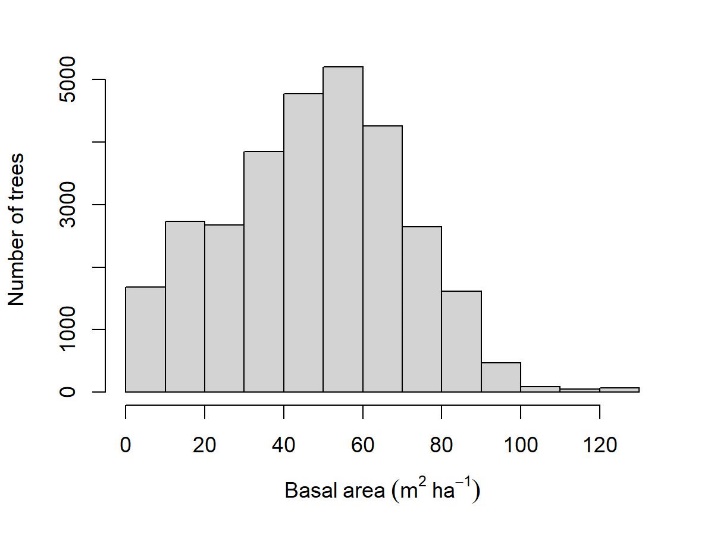

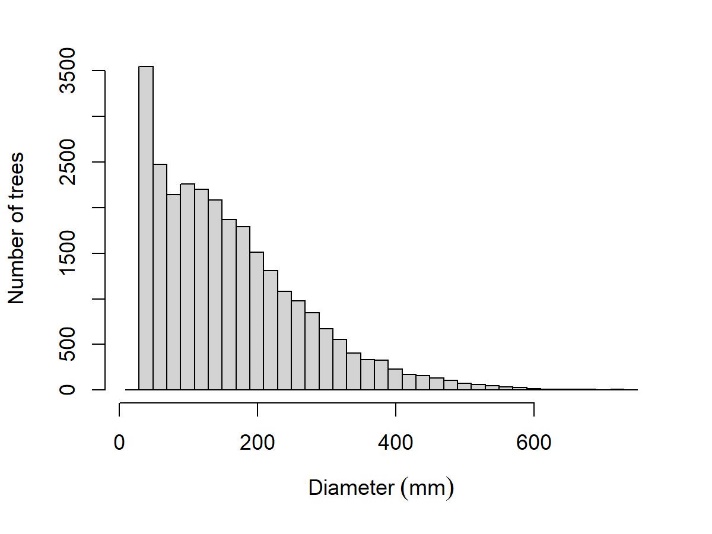
**

**Plot–level**

1. **Soil–available P (b) Landform index**

**
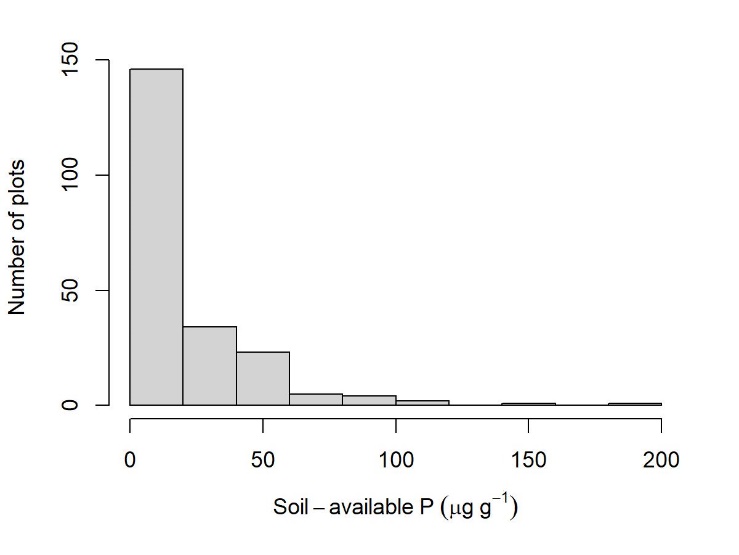

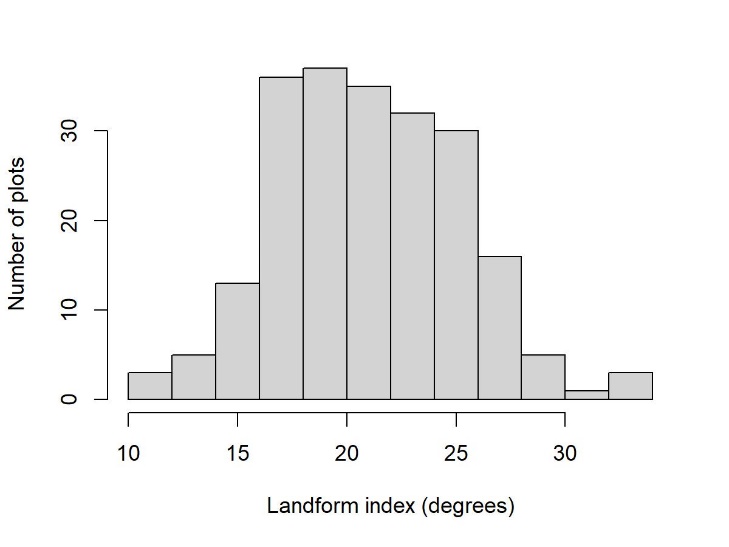
**

1. **Distance from the epicentre (f) Elevation**

**
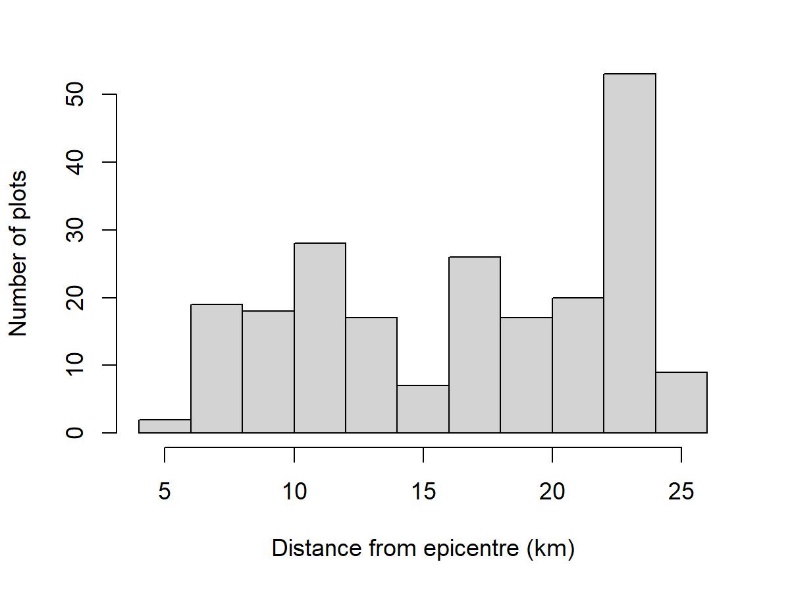

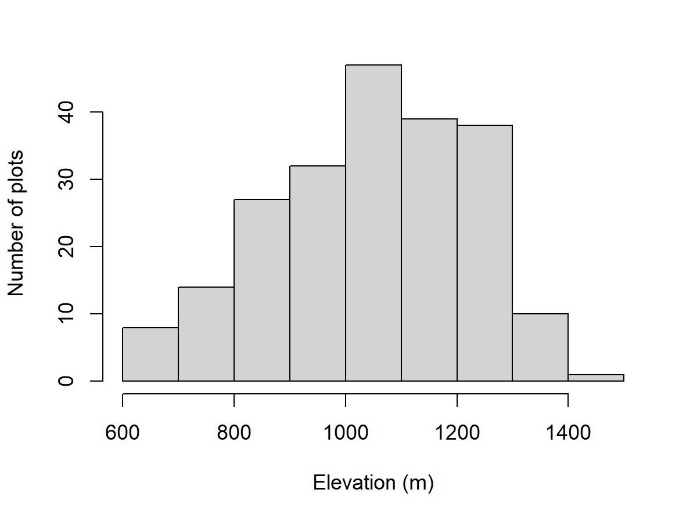
**

**Fig. S3.** Histograms of number of trees (includes re-measurements) and plots (total of 216) for tree– and plot–level covariates respectively, in classes. Tree–level covariates include local basal area (a) and diameter (b). Plot–level covariates include soil–available P (c), landform index (d), distance from the epicentre (e), and elevation (f).

**Appendix S2:** Growth model description and analysis

Following Allen et al (2020), models were developed to describe the growth of individual trees as function of potential covariates. Here we focus on the growth component of our modelling as a way of generating appropriate imputed values for correcting extremes of growth or missing values. The list of tree– and plot–level covariates used here were those suggested by previous literature and were slightly different to those used for survival modelling (see Table 1 versus Appendix S2: Table S1). Fewer plots were used than by Allen et al. (2020). We also included the interaction between basal area and elevation to test whether the effect of neighbourhood competition on growth varied along the stress gradient imposed by elevation. A key aspect of this analysis is to allow the value of the model coefficients to vary over time, in accordance with the three defined survival periods: pre-earthquake, 0–5 years post-earthquake, and 5+ years post-earthquake. A change in the value of a model coefficient over time would reflect changes in the nature of the relationship between the covariate and growth.

**Table S1.** Standardised scale used for each tree– and plot–level covariate employed to model growth, and corresponding units for interpretation of estimated effect size as well as a covariate description

| Covariate | Zero on standardised scale: | Units | Description |
| --- | --- | --- | --- |
| Tree–level |  |  |  |
| $bas_{i,t-1}$ | 50 | m^2^ ha^-1^ | Basal area |
| $y_{i,t-1}$ | 164 | mm | Diameter at breast height |
| Plot–level |  |  |  |
| $avP_{j}$ | exp(2.7) = 14.88 | μg g^-1^ | Soil–available P |
| $LI_{j}$ | 20 | Degrees | Landform index |
| $Sl_{j}$ | 30 | Degrees | Slope |
| $dist_{j}$ | exp(2.9) = 18.17 | ln(km) | Distance from the epicentre |
| $ele_{j}$ | 1000 | m | Elevation |

Bayesian methods of inference were used to estimate model parameters, using the software JAGS from within the R statistical computer environment. Three MCMC chains were run for 150 000 iterations following a burn–in period of 10 000, with every 3^rd^ value being retained. Therefore, inferences were based on posterior distributions approximated from 150 000 samples.

Growth model

The observed size of tree *i* at time *t* ($y_{i,t}$) was defined to be normally distributed with mean ($\mu_{i,t}$) equal to:

$\mu_{i,t}=y_{i,t-1}+\lambda_{i,t}\Delta t$,

and variance $\sigma_{\lambda}^{2}$, where $\lambda_{i,t}$ = growth per year (between times *t*–1 and *t*), and $\Delta t$ is the number of years between times *t*–1 and *t*. Annual growth was modelled as a combination of plot–level effects ($P_{\lambda,j}$), the standardised covariates tree diameter size ($y$) and basal area (*bas*) at time *t*–1 (i.e., conditions at the beginning of the growth period), an interaction between basal area and elevation (*ele*), and a random tree–level effect ($\epsilon_{i}$).

$$\lambda_{i,t}=P_{\lambda,j,t}+\beta_{y,eq}y_{i,t-1}+\beta_{bas,eq}bas_{i,t-1}+\beta_{bas\times ele,eq}bas_{i,t-1}\times ele_{j}+\epsilon_{i}$$

Plot–level effects for tree growth included an overall mean growth rate ($\mu_{\lambda}$), the standardised plot–level covariates soil–available phosphorous (*avP*), landform index (*LI*), distance from the epicentre (*dist*), elevation (*ele*) and slope (*Sl*), and a random plot–level effect ($\epsilon_{j}$).

$$P_{\lambda,j,t}=\mu_{\lambda,eq}+\beta_{avP,eq}avP_{j}+\beta_{LI,eq}LI_{j}+\beta_{dist,eq}dist_{j}+\beta_{ele,eq}ele_{j}+\beta_{Sl,eq}Sl_{j}+\epsilon_{j}$$

Random tree– and plot–level effects were assumed to be normally distributed with mean = 0 and variance $\sigma_{tree}^{2}$ and $\sigma_{plot}^{2}$, respectively. All covariates were standardised to improve the convergence properties of the MCMC chains (Table S1). $\beta_{X,eq}$ represents the coefficient for covariate $X$, in earthquake period *eq*, that is to be estimated.

In summary, the stochastic elements of the growth model is defined as:

$$y_{i,t} N\left( \mu_{i,t},\sigma_{\lambda}^{2} \right)$$

$$\epsilon_{i} N\left( 0,\sigma_{tree}^{2} \right)$$

$$\epsilon_{j} N\left( 0,\sigma_{plot}^{2} \right)$$

Two growth models were fit to the data: one in which the posterior distribution of parameters were estimated simultaneously with the selected survival model (see Appendix S2: Table S2) and one in which the posterior distribution of parameters were estimated simultaneously with the top–ranked survival model (see Appendix S2: Table S3).

**Table S2.** Summary of posterior distributions (PD) for the growth (mm yr^-1^) model parameters estimated in concert with the selected survival model. Parameters include those for the overall model as well as those for tree– and plot–level covariates. A description is given of each parameter followed by the mean, standard deviation (SD), and percentile values of model coefficients for the PD. Parameter values are for the point where other covariates in the model are zero on their standardised scales (Appendix S2: Table S1). As there was little correlation between covariates, the effect will be the same when other standardised covariates are not zero, but the magnitude will be different.

|  |  |  |  | Percentile values | |
| --- | --- | --- | --- | --- | --- |
| Parameter | Description | Mean | SD | 2.5% | 97.5% |
| Overall |  |  |  |  |  |
| $\mu_{\lambda,1}$ | Mean growth pre-earthquake | 1.380 | 0.042 | 1.298 | 1.463 |
| $\mu_{\lambda,2}$ | Mean growth 0–5 years post-earthquake | 1.305 | 0.047 | 1.214 | 1.397 |
| $\mu_{\lambda,3}$ | Mean growth 5+ years post-earthquake | 1.173 | 0.045 | 1.086 | 1.261 |
| $\sigma_{\lambda}$ | SD of random error on observed growth | 1.785 | 0.011 | 1.764 | 1.807 |
| $\sigma_{plot}$ | SD of plot–level random effect | 0.463 | 0.020 | 0.425 | 0.502 |
| $\sigma_{tree}$ | SD of tree–level random effect | 0.640 | 0.009 | 0.624 | 0.657 |
| Tree–level |  |  |  |  |  |
| $\beta_{y,1}$ | Pre-earthquake effect of diameter | 0.00499 | 0.00015 | 0.00469 | 0.00528 |
| $\beta_{y,2}$ | 0–5 years post-earthquake effect of diameter | 0.00459 | 0.00020 | 0.00419 | 0.00499 |
| $\beta_{y,3}$ | 5+ years post-earthquake effect of diameter | 0.00328 | 0.00018 | 0.00293 | 0.00363 |
| $\beta_{bas,1}$ | Pre-earthquake effect of basal area | -0.02521 | 0.00132 | -0.02781 | -0.02261 |
| $\beta_{bas,2}$ | 0–5 years post-earthquake effect of basal area | -0.00801 | 0.00176 | -0.01144 | -0.00456 |
| $\beta_{bas,3}$ | 5+ years post-earthquake effect of basal area | -0.01021 | 0.00147 | -0.01309 | -0.00734 |
| $\beta_{bas\times ele,1}$ | How the pre-earthquake effect of basal area changes with elevation | 0.00498 | 0.00070 | 0.00361 | 0.00634 |
| $\beta_{bas\times ele,2}$ | How the 0–5 years post-earthquake effect of basal area changes with elevation | 0.00028 | 0.00097 | -0.00162 | 0.00218 |
| $\beta_{bas\times ele,3}$ | How the 5+ years post-earthquake effect of basal area changes with elevation | -0.00087 | 0.00081 | -0.00245 | 0.00071 |
| Plot–level |  |  |  |  |  |
| $\beta_{avP,1}$ | Pre-earthquake effect of soil–available P | 0.02835 | 0.04505 | -0.05960 | 0.11689 |
| $\beta_{avP,2}$ | 0–5 years post-earthquake effect of soil–available P | 0.00496 | 0.04891 | -0.09092 | 0.10088 |
| $\beta_{avP,3}$ | 5+ years post-earthquake effect of soil–available P | 0.13352 | 0.04672 | 0.04270 | 0.22556 |
| $\beta_{LI,1}$ | Pre-earthquake effect of landform index | -0.01031 | 0.00967 | -0.02935 | 0.00869 |
| $\beta_{LI,2}$ | 0–5 years post-earthquake effect of landform index | 0.01907 | 0.01094 | -0.00224 | 0.04050 |
| $\beta_{LI,3}$ | 5+ years post-earthquake effect of landform index | 0.00881 | 0.01050 | -0.01181 | 0.02941 |
| $\beta_{ele,1}$ | Pre-earthquake effect of elevation | -0.10339 | 0.02397 | -0.15025 | -0.05616 |
| $\beta_{ele,2}$ | 0–5 years post-earthquake effect of elevation | -0.07926 | 0.02724 | -0.13358 | -0.02597 |
| $\beta_{ele,3}$ | 5+ years post-earthquake effect of elevation | -0.08093 | 0.02578 | -0.13151 | -0.03044 |
| $\beta_{dist,1}$ | Pre-earthquake effect of distance from epicentre | -0.350 | 0.094 | -0.537 | -0.166 |
| $\beta_{dist,2}$ | 0–5 years post-earthquake effect of distance from epicentre | 0.076 | 0.103 | -0.125 | 0.278 |
| $\beta_{dist,3}$ | 5+ years post-earthquake effect of distance from epicentre | -0.282 | 0.099 | -0.476 | -0.089 |
| $\beta_{Sl,1}$ | Pre-earthquake effect of slope | 0.144 | 0.346 | -0.533 | 0.822 |
| $\beta_{Sl,2}$ | 0–5 years post-earthquake effect of slope | -0.445 | 0.371 | -1.174 | 0.281 |
| $\beta_{Sl,3}$ | 5+ years post-earthquake effect of slope | 0.085 | 0.360 | -0.619 | 0.796 |

**Appendix S3:** Summary of posterior distributions of top–ranked survival model.

**Table S1.** Summary of posterior distributions (PD) of parameters for the top–ranked survival model (on logit scale). Parameters include those for the overall model as well as those for tree– and plot–level covariates. A description is given of each parameter followed by the mean, standard deviation (SD), and percentile values for the PD. Parameter values are for the point where other covariates in the model are zero on their standardised scales (Table 1). As there was little correlation between covariates, the effect will be the same when other standardised covariates are not zero, but the magnitude will be different.

|  |  |  |  | Percentile values | |
| --- | --- | --- | --- | --- | --- |
| Parameter | Description | Mean | SD | 2.5% | 97.5% |
| Overall |  |  |  |  |  |
| $\mu_{S,1}$ | Mean survival pre-earthquake (logit scale) | 3.653 | 0.129 | 3.402 | 3.908 |
| $\mu_{S,2}$ | Mean survival 0–5 years post-earthquake (logit scale) | 3.914 | 0.164 | 3.598 | 4.240 |
| $\mu_{S,3}$ | Mean survival 5+ years post-earthquake (logit scale) | 3.638 | 0.137 | 3.372 | 3.911 |
| $\sigma_{S}$ | SD of plot–level random effect | 0.967 | 0.060 | 0.853 | 1.089 |
| Tree–level |  |  |  |  |  |
| $\gamma_{y,1}$ | Pre-earthquake linear effect of diameter | 0.00481 | 0.00076 | 0.00332 | 0.00632 |
| $\gamma_{y^{2},1}$ | Pre-earthquake quadratic effect of diameter | -0.00002 | 0.00000 | -0.00003 | -0.00002 |
| $\gamma_{y,2}$ | 0–5 years post-earthquake linear effect of diameter | 0.00550 | 0.00100 | 0.00354 | 0.00748 |
| $\gamma_{y^{2},2}$ | 0–5 years post-earthquake quadratic effect of diameter | -0.00002 | 0.00000 | -0.00003 | -0.00001 |
| $\gamma_{y,3}$ | 5+ years post-earthquake linear effect of diameter | 0.00196 | 0.00085 | 0.00029 | 0.00362 |
| $\gamma_{y^{2},3}$ | 5+ years post-earthquake quadratic effect of diameter | -0.00001 | 0.00000 | -0.00002 | -0.00001 |
| $\gamma_{bas,1}$ | Pre-earthquake effect of basal area | -0.010 | 0.005 | -0.020 | 0.000 |
| $\gamma_{bas,2}$ | 0–5 years post-earthquake effect of basal area | -0.002 | 0.006 | -0.013 | 0.009 |
| $\gamma_{bas,3}$ | 5+ years post-earthquake effect of basal area | 0.002 | 0.005 | -0.007 | 0.011 |
| $\gamma_{bas\times ele,1}$ | How the pre-earthquake effect of basal area changes with elevation | -0.004 | 0.002 | -0.009 | 0.000 |
| $\gamma_{bas\times ele,2}$ | How the 0–5 years post-earthquake effect of basal area changes with elevation | 0.009 | 0.003 | 0.003 | 0.015 |
| $\gamma_{bas\times ele,3}$ | How the 5+ years post-earthquake effect of basal area changes with elevation | -0.005 | 0.003 | -0.010 | 0.000 |
| $\gamma_{G,1}$ | Pre-earthquake effect of previous growth | 0.544 | 0.067 | 0.413 | 0.678 |
| $\gamma_{G,2}$ | 0–5 years post-earthquake effect of previous growth | 0.215 | 0.063 | 0.091 | 0.340 |
| $\gamma_{G,3}$ | 5+ years post-earthquake effect of previous growth | 0.754 | 0.075 | 0.608 | 0.903 |
| $\gamma_{G\times ele,1}$ | How the pre-earthquake effect of previous growth changes with elevation | -0.029 | 0.031 | -0.091 | 0.032 |
| $\gamma_{G\times ele,2}$ | How the 0–5 years post-earthquake effect of previous growth changes with elevation | 0.042 | 0.037 | -0.032 | 0.114 |
| $\gamma_{G\times ele,3}$ | How the 5+ years post-earthquake effect of previous growth changes with elevation | 0.136 | 0.039 | 0.058 | 0.212 |
| $\gamma_{G1,1}$ | Pre-earthquake effect of lagged growth | 0.462 | 0.069 | 0.327 | 0.598 |
| $\gamma_{G1,2}$ | 0–5 years post-earthquake effect of lagged growth | 0.229 | 0.073 | 0.088 | 0.373 |
| $\gamma_{G1,3}$ | 5+ years post-earthquake effect of lagged growth | 0.327 | 0.062 | 0.206 | 0.450 |
| $\gamma_{G1\times ele,1}$ | How the pre-earthquake effect of lagged growth changes with elevation | -0.083 | 0.033 | -0.147 | -0.018 |
| $\gamma_{G1\times ele,2}$ | How the 0–5 years post-earthquake effect of lagged growth changes with elevation | 0.045 | 0.041 | -0.036 | 0.124 |
| $\gamma_{G1\times ele,3}$ | How the 5+ years post-earthquake effect of lagged growth changes with elevation | -0.035 | 0.033 | -0.101 | 0.030 |
| Plot–level |  |  |  |  |  |
| $\gamma_{avP,1}$ | Pre-earthquake effect of soil–available P | -0.052 | 0.117 | -0.282 | 0.178 |
| $\gamma_{avP,2}$ | 0–5 years post-earthquake effect of soil–available P | -0.221 | 0.132 | -0.480 | 0.037 |
| $\gamma_{avP,3}$ | 5+ years post-earthquake effect of soil–available P | 0.071 | 0.120 | -0.164 | 0.307 |
| $\gamma_{LI,1}$ | Pre-earthquake effect of landform index | -0.009 | 0.023 | -0.054 | 0.036 |
| $\gamma_{LI,2}$ | 0–5 years post-earthquake effect of landform index | -0.093 | 0.025 | -0.142 | -0.044 |
| $\gamma_{LI,3}$ | 5+ years post-earthquake effect of landform index | -0.026 | 0.024 | -0.074 | 0.022 |
| $\gamma_{ele,1}$ | Pre-earthquake effect of elevation | 0.272 | 0.066 | 0.143 | 0.402 |
| $\gamma_{ele,2}$ | 0–5 years post-earthquake effect of elevation | -0.044 | 0.089 | -0.220 | 0.130 |
| $\gamma_{ele,3}$ | 5+ years post-earthquake effect of elevation | 0.054 | 0.073 | -0.088 | 0.196 |
| $\gamma_{dist,1}$ | Pre-earthquake effect of distance from epicentre | -0.043 | 0.237 | -0.510 | 0.417 |
| $\gamma_{dist,2}$ | 0–5 years post-earthquake effect of distance from epicentre | 1.039 | 0.252 | 0.544 | 1.533 |
| $\gamma_{dist,3}$ | 5+ years post-earthquake effect of distance from epicentre | 0.002 | 0.249 | -0.485 | 0.491 |
|  |  |  |  |  |  |
| Deviance |  | 135725.931 | 184.941 | 135365.622 | 136091.609 |
